# Implementation of the Ottawa Hospital Pain Clinic stepped care program: A preliminary report

**DOI:** 10.1080/24740527.2020.1768059

**Published:** 2020-08-13

**Authors:** Louise Bell, Peter Cornish, Renée Gauthier, Cristin Kargus, Joshua Rash, Rose Robbins, Susan Ward, Patricia A. Poulin

**Affiliations:** a Department of Psychology, Memorial University of Newfoundland, St. John’s, Newfoundland, Canada; b Student Wellness & Counselling Centre, Memorial University of Newfoundland, St. John’s, Newfoundland, Canada; c The Ottawa Hospital Pain Clinic, Ottawa, Ontario, Canada; d Department of Anesthesiology and Pain Medicine, Faculty of Medicine, University of Ottawa, Ottawa, Ontario, Canada

**Keywords:** chronic pain, pain management, interprofessional treatment, stepped care

## Abstract

**Background:**

Access to multidisciplinary pain management treatment in Canada is limited, with wait times up to 4 years. Stepped care approaches to mental health treatment have led to substantial reduction and elimination of wait times and may be applicable to chronic pain settings. There is no unifying framework for stepped care chronic pain programs. A systematic review of the efficacy of stepped care in chronic pain management conducted by the Canadian Agency for Drugs and Technologies reported varied results that may be due to heterogeneous stepped care models across facilities.

**Aim:**

We propose a unifying framework for multidisciplinary stepped care chronic pain programs and present its application at The Ottawa Hospital Pain Clinic. The Ottawa Hospital stepped care framework is an eight-tiered approach that allows patients the opportunity to decide collaboratively with a health care professional which treatment program will best suit their needs for the management of chronic pain. As levels of stepped care increase, the time and resource commitment to each step will also increase. Treatment is stepped up or down, depending on patient needs.

**Method:**

This is a descriptive case study.

**Results:**

Implementing the interprofessional model of care with the stepped care program has eliminated wait times for access to The Ottawa Hospital Pain Clinic Interprofessional Chronic Pain Management Program and has improved communication between professions of the interprofessional team, resulting in better care for patients.

**Conclusion:**

More research is needed to further develop and evaluate the clinical efficacy of stepped care to manage chronic pain.

## RÉSUMÉ

**Contexte**: L’accès à la prise en charge multidisciplinaire de la douleur au Canada est limité, avec des délais d’attente pouvant aller jusqu’à quatre ans. Les approches de soins de santé mentale par paliers ont donné lieu à une réduction et une élimination des temps d’attente et peut être applicable aux contextes de soins pour la douleur chronique. Il n’existe pas de cadre unificateur pour les programmes de soins par paliers pour la douleur chronique. Un examen systématique de l’efficacit’ des soins par paliers dans la prise en charge de la douleur chronique menée par l’Agence canadienne des médicaments et des technologies de la santé a fait état de résultats variés qui peuvent être attribuables à l’hétérogénéité des modèles de soins par paliers dans les vtablissements.

**Objectifs**: Nous proposons un cadre unificateur pour les programmes de soins multidisciplinaires par paliers pour la douleur chronique et présentons son application à la Clinique de la douleur de l’Hôpital d’Ottawa. Le cadre de soins par paliers de l’Hôpital d’Ottawa est une approche à huit niveaux qui donne aux patients la possibilité de décider, en collaboration avec un professionnel de la santé, du programme de traitement qui répondra le mieux à leurs besoins pour la prise en charge de leur douleur chronique. À mesure que les niveaux de soins par paliers augmentent, le temps et les ressources nécessaires à chaque palier augmentent également. Le traitement est intensifié ou réduit, en fonction des besoins du patient.

**Méthodes**: Il s’agit d’une étude de cas descriptive.

**Résultats**: La mise en œuvre du modèle interprofessionnel de soins avec le programme de soins par paliers a éliminé les délais d’attente pour l’accès au programme de prise en charge interprofessionnelle de la douleur chronique de la clinique de la douleur de l’Hôpital d’Ottawa et a amélioré la communication entre les professions de l’équipe interprofessionnelle, ce qui a donné lieu à une meilleure prise en charge des patients.

**Conclusions**: Des recherches supplémentaires sont nécessaires pour développer et évaluer davantage l’efficacité clinique des soins par paliers pour la prise en charge de la douleur chronique.

## Introduction

### Prevalence of Chronic Pain

Chronic pain is defined by the International Association for the Study of Pain as “an aversive sensory and emotional experience typically caused by, or resembling that caused by, actual or potential tissue injury.”^1^ The World Health Organization incorporated chronic pain as a chronic disease in May 2019 in the *International Classification of Diseases* (11th Revision).^2,3^ Chronic pain affects approximately one in every five Canadians, or about 6 million Canadians of all ages.^4^ A national survey reported that more than 50% of those affected have had chronic pain for more than 10 years and report having moderate to severe pain.^4^

In addition to the human impact, chronic pain presents a significant financial burden. Hogan and colleagues^5^ conducted a case–control study using data from the Canadian Community Health Survey and reported that the annual cost per person for health care among patients with chronic pain living in Ontario was CAD5177, which was CAD1742 greater than costs for matched patients without chronic pain. A national study demonstrated that patients incur an estimated cost of CAD1462 per month to cover out-of-pocket expenses, such as medications and health care services not covered by insurance plans.^6^ The total annual burden of chronic pain on the Canadian health care system is estimated at CAD7.2 billion adjusted to 2014 Canadian dollars.^5^

### Best Practices and Accessibility

Chronic pain is best understood through a biopsychosocial lens taking into account the complex interaction between physiological, psychological, and social factors that influence the experience of pain.^7^ This multidimensional model is often used to inform the treatment of chronic pain.^8,9^ Multidisciplinary pain management using a biopsychosocial approach is considered the gold standard.^8,10^ To be considered a multidisciplinary program, the program must be delivered with at least two different health care specialties, including medicine, nursing, psychology, social work, physiotherapy, and/or occupational therapy.^11,12^ The multidisciplinary pain management team members are often located within the same clinic, with frequent contact through team meetings to discuss unified goals and values for the program and for patients.^11^ Extensive research has been conducted establishing both treatment efficacy and cost-effectiveness of multidisciplinary programs for the management of chronic pain.^6,10,13^

At present, multidisciplinary pain centers have protracted wait lists as long as 4 years.^14^ Lengthy wait times can often result in health consequences, which can subsequently impact psychological well-being and functioning for people with chronic pain. Individuals who wait more than 6 months to receive care often experience a significant deterioration in their quality of life.^15^ For this reason, wait times longer than 6 months are considered medically and ethically unacceptable.^16^ A potential solution to improving wait times is the implementation of a stepped care framework. Chronic pain care teams can learn from the application and success of stepped care in mental health settings.

### Stepped Care in a Mental Health Setting

Demand for mental health services in Canada far exceeds available resources.^17^ As a result, individuals with mental health care needs in Canada experience difficulties in accessing appropriate and timely care that are similar to those experienced among individuals with chronic pain. The Mental Health Commission of Canada reviewed the state of mental health care in 2012 and concluded that Canada is lacking a system to organize the receipt of mental health care that is accessible, organized, and effective.^18^ The Mental Health Commission of Canada recommended the establishment of an efficient system to provide Canadians with early and rapid assessment, as well as systematic access, to the most effective treatment, where and when they need it.

Stepped care was originally developed to improve efficiency in the delivery of primary care in the United Kingdom and has been reimagined to maximize the effectiveness of and access to mental health services.^17^ The model advocates for the lowest level of intervention intensity warranted by the initial assessment and stepping up or down therapy based on treatment response, patient preference, motivation, preparedness, distress, or needs.^19,20^ The model also requires ongoing monitoring to inform these treatment decisions.^21,22^

Empowerment is an integral component of stepped care. The World Health Organization defines empowerment as “a process through which people gain greater control over decisions and actions affecting their health”^23^ and should be viewed as an individual and community process. Involvement in decisions about one’s care can facilitate perceived and actual control. Stepped care allows the individual and health care providers to work collaboratively to meet the needs of the client, which could involve stepping up or down therapy intensity to achieve the health goals agreed upon by the dyad.

**Figure 1.**
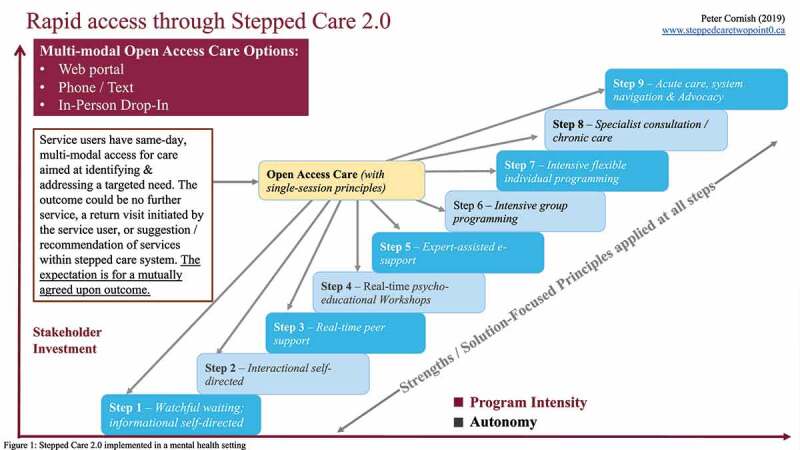
Stepped Care 2.0 implemented in a mental health setting

Cornish and colleagues^17^ adapted and implemented a reimagined version of stepped care (Stepped Care 2.0) in mental health settings across Newfoundland and Labrador, Canada.^20^ Stepped Care 2.0 (Figure 1) typically consists of nine steps (though steps are tailored to local context and the number may vary depending on jurisdiction and availability of resources), including self-help, peer support, Internet-based programs, same-day access to mental health care, and specialist care.^17^ Following the implementation of Stepped Care 2.0 across Newfoundland and Labrador in 2017–2018, wait times to access mental health and addiction services were reduced by 68%.^20^

### Stepped Care in Multidisciplinary Chronic Pain Settings

Given the success of stepped care in mental health settings and given the similar needs for education, self-management, and support found among people living with mental health difficulties and people living with chronic pain, a stepped care approach may be part of the solution to reduce wait times for chronic pain care and increase patient empowerment, thereby reducing the burden of chronic pain for people and society.

Similar to Stepped Care 2.0^17^ delivered in the mental health setting, the adoption of this model in chronic pain management settings uses the least intensive interventions that meet the patient’s needs, taking into account the evidence supporting different interventions, patient preference, and patient readiness.^24^ Implementing a comprehensive stepped care framework in a chronic pain setting incorporates and expands on the work of Cornish et al.^17^ It leverages local and online resources as well as the expertise of peers and providers from different disciplines with specialized training in chronic pain management to address patients’ needs and assist them in achieving their goals.

The Canadian Agencies for Drugs and Technologies in Health (CADTH) conducted a systematic review evaluating the clinical effectiveness of stepped care programs that were all introduced in multidisciplinary pain clinics.^21^ Of the 12 studies that met inclusion criteria, a total of nine articles (including two systematic reviews) specifically evaluated the efficacy of stepped care implemented in a pain clinic setting.

Two systematic reviews of randomized controlled trials (RCTs) were considered to have a low risk of bias and ranged from low to good quality of evidence.^25–27^ Peterson et al.^25^ identified four RCTs that reported on interventions that included specific stepped care components and concluded that stepped care resulted in improvement in pain and function at 9 to 12 months posttreatment. The strength of this evidence was judged “low” by CADTH because the effect of stepped care on pain and function outcomes was only supported by a single RCT with imprecise results. Similarly, Cochrane et al.^27^ included 20 RCTs that evaluated return-to-work and work absence outcomes, of which 4 evaluated the effects of stepped care at 12 months postintervention. Low-quality evidence from four RCTs indicated that stepped care programs were reportedly more effective than comparators at improving return to work (hazard ratio = 1.29; 95% confidence interval, 1.03–1.61). Imprecision was a concern, with substantial between-trial heterogeneity (*I*^2^ = 50%).

One additional RCT^28^ and six nonrandomized controlled trials^19,24,29–32^ that were considered to have a high risk of bias were published since the time the initial reviews met inclusion criteria. Models varied across the United Kingdom and United States, demonstrated inconsistencies in the implementation of stepped care, and included a maximum of three steps to the model. None included Internet-based interventions.

Of the nine studies using stepped care, two studies^24,30^ utilized the model developed by the Department of Veterans Affairs,^33^ which was the most consistent with stepped care guidelines to date. This model is a three-step approach that includes a variety of professionals, including primary care providers, nurse practitioners, nurses, clinical psychologists, and physiotherapists.^33^ The first step provides psychoeducation and self-care information to the patient and family in addition to screening and assessment of the presence and severity of pain from a primary care medical team. Step 2 includes a secondary consultation, which provides care from multidisciplinary pain medicine specialty teams. Individuals would receive a variety of interventions, such as behavioral pain management, access to rehabilitation medicine, and/or mental health/substance use disorder programs.^33^ Finally, step 3 employs the most advanced care by providing patients with advanced pain medicine diagnostics and interventions in addition to access to the Commission of Accreditation of Rehabilitation Facilities pain rehabilitation.^33^ The clinical outcomes of these two studies were mixed, with one demonstrating improved pain management and the other demonstrating poorer pain control among patients who were enrolled in the programs. Other studies often incorporated decision support algorithms or focused on a limited number of interventions and did not adhere directly to the deliverance of a formalized stepped care model.^21^

Overall, CADTH reported that the effects of stepped care on pain and function among individuals with chronic pain were equivocal with some,^19,25,29^ but not all,^28,32^ studies reporting positive effects. Evidence from the inconsistent findings confirm that data is limited, and additional rigorous trials are required that more clearly outline the model of stepped care being delivered, along with a careful identification of steps, stepping algorithms, and implementation. This, in turn, will help to determine the clinical efficacy of stepped care for the management of chronic pain and can better inform treatment decisions when using stepped care for the management of chronic pain.^21^ The purpose of this article is to describe the development of a comprehensive chronic pain stepped care program modeled on Stepped Care 2.0^17^ in a tertiary pain clinic in Canada. This study will add to the growing literature surrounding the implementation of stepped care for people with chronic pain, which can consequently help to identify best practice for implementing a stepped care approach in chronic pain settings.

## Stepped Care to Manage Chronic Pain in a Tertiary Pain Clinic Setting

The Ottawa Hospital is an urban tertiary academic medical center located in Ontario, Canada. The pain clinic is staffed by anesthesiologists, one physiatrist, one pain medicine specialist, nurses, and an interprofessional team consisting of two psychologists, one social worker, one physiotherapist, and one occupational therapist. The pain clinic is one of 17 clinics that have received funding from the Ontario Ministry of Health and Long-Term Care to help alleviate the burden of chronic pain in Ontario.

**Figure 2.**
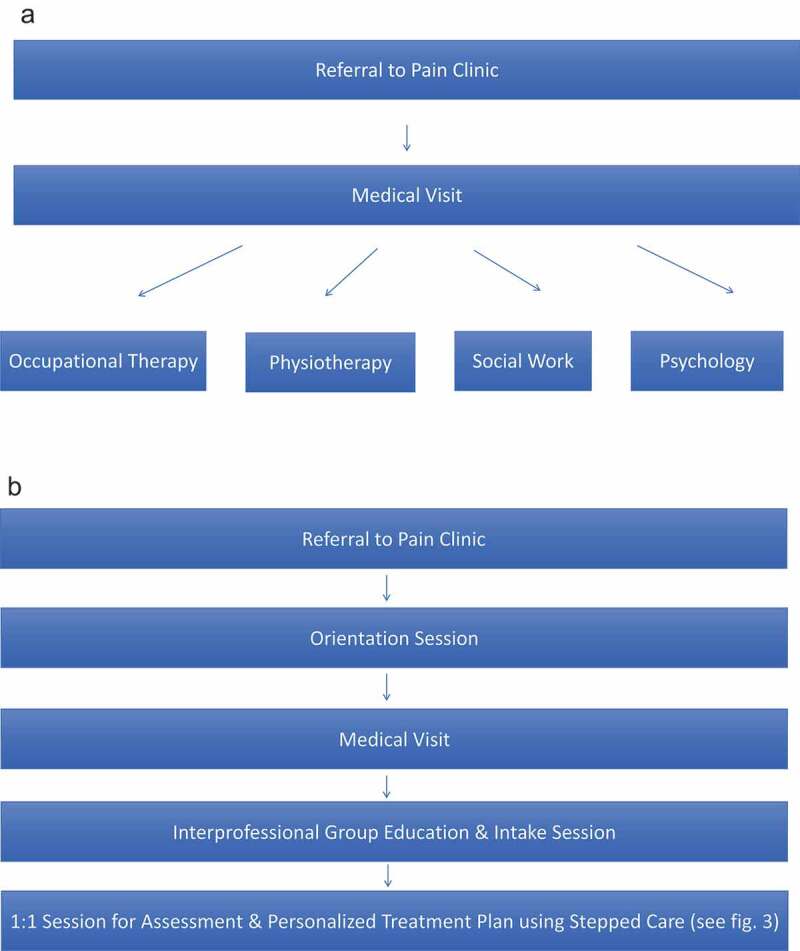
(a) Old referral pathway. (b) New referral pathway

Until 2017, in line with many pain management clinics, the pain clinic at the Ottawa Hospital General Campus followed a multidisciplinary biopsychosocial approach to the management of chronic pain. More recently, the pain clinic moved from a multidisciplinary framework to an interprofessional model of care using the same staffing model and developed a stepped care framework to improve access to its various services and programs. Historically, physicians within The Ottawa Hospital Pain Clinic would refer their patients to one or several of four disciplines: occupational therapy, physiotherapy, social work, or psychology (see Figure 2a), who would be operating relatively independently from one another, with the exception of routine case conferences for patients presenting with complex needs. Referrals quickly outpaced capacity for treatment, resulting in an ever-increasing wait time to access their individualized programming. Health care professionals from any given discipline also often identified additional needs for their patients during patient assessment that required attention from other professionals on the team. This further increased the waiting period and resulted in disjointed care. Within 6 months of having reached full staffing status, the estimated wait time for physiotherapy and psychology grew to an estimated 6 months.

**Figure 3.**
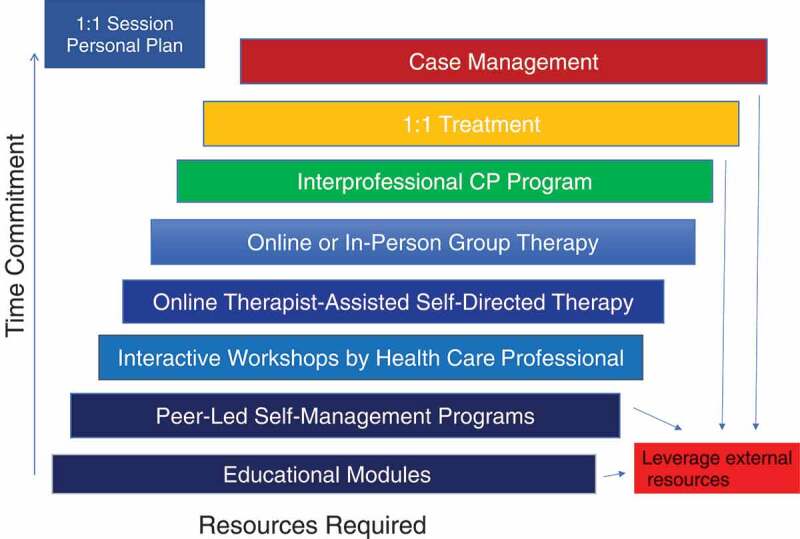
The Ottawa Hospital Pain Clinic eight-tiered interprofessional chronic pain management stepped care framework

Informed by the work of Cornish and colleagues,^17^ the team developed a stepped care interprofessional chronic pain management program integrating a 90-min orientation session for all patients referred to the pain clinic. The orientation session is delivered weekly by one of the psychologists and the social worker from the interprofessional team. Patients are provided with psychoeducation on chronic pain, programs and opportunities available at the clinic, and community resources that are offered outside the hospital (see Table 1). This session allows for patients to become potentially more ready to engage with programs available at the clinic if deemed appropriate after their physician appointment. It is also designed to engage clients to move along the stages of change model (e.g., from pre-contemplation to contemplation), because individuals who are further along the stages of change often show better results in treatment outcomes.^34–37^ This is done by encouraging participants to identify goals that are not solely based on pain but are also based on values.^38^ They are additionally provided information about best practices to chronic pain management such as informing treatment decisions based on the biopsychosocial approach and what can be expected from treatment at the pain clinic. This information often helps individuals think differently about their pain and its management.

**Table 1.** Information provided to patients during orientation session at the Ottawa Hospital Pain Clinic

Why a group session?
Definition of pain and difference between acute and chronic pain
Hurt vs. harm in chronic pain
Impact of chronic pain
Biopsychosocial approach to address multiple dimensions of chronic pain
Building your toolkit—What to do above and beyond medical management and setting realistic expectations
Risk–benefit of opioid use in the management of chronic pain
Research vignettes on the impact of “understanding pain” and nonpharmacological management
Next steps: What to expect at your first visit with pain specialist
Programs offered by the interprofessional team
Community resources (psychosocial, legal, financial, medical)

Following the orientation session, patients attend a physician appointment during which the physician assesses the patient and discusses with the patient what medical options might best suits their needs (i.e., change in pharmacotherapy prescription or an interventional approach such as epidural steroid injections, radio-frequency ablation, spinal cord stimulation, etc.). During this session, patients complete a Brief Pain Inventory^39^ and a referral is sent to the interprofessional team if scores pertaining to functional limitations are elevated on the Brief Pain Inventory (e.g., significant insomnia, depression, anxiety, or functional limitations in activities of daily living; see Figure 2b).

Patients referred to the interprofessional team are then scheduled by staff to attend the interprofessional group intake session. This 90-min session is delivered by one member of the team on a rotation basis and provides patients with additional education about chronic pain, approaches to chronic pain management including self-management skills, and information about the workshops and group therapy programs available at the clinic. Patients complete a more comprehensive battery of questionnaires (e.g., Patient Health Questionnaire-9, Generalized Anxiety Disorder Scale-7, Insomnia Severity Index, Pain Catastrophizing Scale, Tampa Scale of Kinesiophobia, Limitations in Daily Activities Scale, Stage of Change questionnaire, goals, social determinants of health-related questions, demographics) during this session to determine their goals and care needs. Following the session, a one-on-one phone, video conference, or in-person appointment is scheduled with the respective team member who delivered the group session to develop a personalized care plan that can be revisited if and when the patient deems it necessary. This plan leverages in-house and community programs and resources, as well as online material/programs, using a stepped care approach.

### The Ottawa Hospital Pain Clinic Stepped Care Program Options

The Ottawa Hospital Pain Clinic Stepped Care Program includes eight tiers that provide access to a variety of interventions and programs to ensure the needs of the patients are met using the least intensive and most appropriate interventions available (see Figure 3). Each step is based on Cornish and colleague’s^17^ stepped care model but modified according to availability of programs within Ontario and taking into account the needs of individuals with chronic pain. The eight steps are arranged from the least to most intensive as follows: (1) online reading/self-directed educational modules; (2) peer-led self-management programs; (3) interactive online or in-person group-based workshops led by health care professionals; (4) online therapist-assisted self-directed therapy; (5) online or in-person group therapy; (6) interprofessional chronic pain rehab program; (7) one-on-one treatment; and (8) complex case management. After meeting with a member of the inter professional team to complete an individual treatment plan, the patient is provided with a written plan that may include one or a combination of interventions. Consistent with stepped care guidelines,^17^ this is not a pathway model where patients must complete a lower intensity intervention prior to being able to access higher intensity treatment. Therapy intensity is stepped up or down as needed considering clinical outcomes and client factors (e.g., preference, self-efficacy, stage of change, motivation), and multiple interventions of different intensity may be combined to address a patient’s needs (e.g., group cognitive–behavioral therapy for anxiety and depression combined with a one-on-one physiotherapy assessment for a specific set of exercises to address failed back surgery syndrome).

#### Step 1

The first step entails online readings and modules. The interprofessional team has developed a list of trusted resources (i.e., Pain BC^40^) for patients that cover pain education, managing mood/anxiety/insomnia, and patient stories of recovery. These are presented to patients as needed.

#### Step 2

The second step provides patients information for peer-led self-management programs. Patients are given information to access these programs, such as the Living Healthy with Chronic Pain program offered in various locations throughout the Ottawa region.^41^

#### Step 3

In-clinic (with online options when appropriate) group workshops are provided for step 3 by the interprofessional team members. Workshops are available 1 to 2 days per week. Some workshops available include disability tax credit provided by the social worker; how to exercise with chronic pain taught by the physiotherapist; ergonomics and body mechanics for everyday life delivered by the occupational therapist; and cognitive–behavioral therapy boosters provided by the psychologist, available only to those who have previously completed the high-intensity, group-based depression and anxiety intervention at the pain clinic. Some workshops are offered via an online video conference platform, for patients who are otherwise unable to travel to the clinic due to physical or geographical barriers. Patients are invited to sign up to any workshop they feel may best suits their needs. The large selection of workshops helps to address the many areas that may be impacted by the experience of pain. It also allows patients to take ownership of their treatment plan as they select the workshops that will provide the education they require according to their self-identified needs. Lastly, workshops provide them with an opportunity to become familiarized with group-based activities, which may be especially beneficial for patients who are hesitant to enroll in group therapy.

#### Step 4

The clinic offers online therapist-assisted self-directed therapy, a mindfulness-based pain management program. This is a video-based program with bi-weekly coaching sessions by one of the psychologists. Patients may also be directed to other targeted online resources such as BounceBack,^42^ a provincial program to help reduce symptoms of depression and anxiety, and Big White Wall,^43^ an online mental health and well-being service that offers various self-help programs.

#### Step 5

Discipline-specific group therapy targeting focal problems patients present with are available at step 5. Group therapies specific to psychology include pelvic pain, online drop-in mindfulness, cognitive–behavioral therapy for insomnia, and a transdiagnostic depression and anxiety group. Physiotherapy group programs include aquatherapy for widespread pain, exercise for pelvic pain, qi gong, and yoga. Occupational therapy groups include mindfulness-based pain management and pacing. Finally, the social work groups include parenting with chronic pain, a social work discussion group, young adults with chronic pain, and a family-focused group for people with chronic pain. Each group facilitator completes a screen or full assessment to ensure that the group is the right fit for each patient.

#### Step 6

An interprofessional chronic pain rehabilitation program called the Low Intensity Treatment and Education (LITE) chronic pain management program is available at step 6. This program consists of eight weekly 3.5-h sessions. Each session allots 1 h for occupational therapy, 1 h for physiotherapy, and 1 h for psychology. Additionally, the social worker will engage in discussion about community resources, assertive communication, and communicating with health care professionals about chronic pain during the seventh week. At the end of the program, patients can be referred for a further 100 h of intensive chronic pain programs at our rehabilitation center facility if they have the interest and tolerance to complete the program.

#### Step 7

Step 7 provides individual therapy for patients. Each discipline has specific one-on-one therapy referral criteria. For example, complex regional pain syndrome and failed back surgery syndrome immediately trigger a one-on-one referral for physiotherapy; suicide risk assessment or psychodiagnostics assessment for people presenting with complex needs triggers a one-on-one referral for psychology; moderate to severe impairment to activities of daily living or a falls risk triggers a one-on-one referral to occupational therapy; and significant concerns relative to social determinants of health triggers a referral to social work. Patients are referred to individual therapy when they require intensive therapy that cannot otherwise be provided by lower intensity steps or if programs available for them at the clinic do not suit their specific needs.

#### Step 8

Step 8 is the most intensive level of care, reserved for patients with the highest level of need, and entails complex case management. The pain clinic has developed a specialized program for patients with chronic pain who have multiple visits to the emergency department.^44–46^ Patients referred to this program generally have complex medical and psychosocial problems that require well-integrated interprofessional care, including medical and case management. When necessary, patients are referred to Health Links^47^ or Primary Care Outreach^48^ for further support.

## Preliminary Impact

Preliminary results from implementing the eight-tiered stepped care model are promising. Services for the interprofessional team have increased as evidenced by the elimination of wait times. Both the transition from a multidisciplinary program to an interprofessional program and implementing one overarching referral rather than four individual referrals have significantly improved access to the interprofessional team. Before the implementation of stepped care, the members of the interprofessional team met weekly and discussed wait times for their respective professional services. Although no tracking system was in place that allowed for a precise estimate, wait times were as long as 6 months for access to specific professionals before the implementation of stepped care. A tracking system was established through a spreadsheet accessible to all members of the team as part of the streamlined referral process and implementing stepped care. The patient’s date of referral, date contacted by the pain clinic, outcome of the first contact (i.e., deciding whether to participate in the interprofessional program), date of completion of the interprofessional group session, and the completion of the one-on-one meeting with an interprofessional member were tracked. Specifically, patients were contacted on average within 2 days of their referral to the team to schedule the group intake appointment, and 90% of patients completed the assessment process within one month. This approach has also led to increased interprofessional collaborations and improved care for patients: The interprofessional team meets weekly to discuss specific cases and/or plans of improvement for patient care. The systematic approach to patient education and assessment also allows for a consistent message about pain management across all the disciplines. In addition to shorter wait times, the adoption of a group treatment approach has increased efficiencies such that more patients can be followed longer by each professional. Finally, group treatments help foster connections between patients who may often feel isolated by their pain condition.

It is also necessary to rigorously evaluate the program and involve patients in redesign in order to ensure that care for patients is continuously improving. The team is routinely collecting pre/post data for all interprofessional groups and is currently conducting formal evaluation of the orientation session, the 8-week Low Intensity Treatment and Education Program, the Pelvic Pain Program, the Mindfulness-Based Pain Management program, and the transdiagnostic Cognitive–Behavioral Therapy for Anxiety and Depression Program. Results from the evaluations will provide information for next steps to improve the quality of each program for patients. We are also in the process of examining whether expressed/identified patient needs (e.g., improvement of sleep) are matched with the delivery of care (e.g., providing the cognitive–behavioral therapy for insomnia group for the patient).

## Future Considerations

The implementation of stepped care is relatively novel, and there is room for growth within the program. For example, in Canada, most chronic pain programs are designed to treat patients who are prepared to accept treatment recommendations immediately and make the difficult lifestyle changes prescribed by practitioners despite the observation that a considerable proportion of patients with chronic pain are not ready to make a concerted action toward change.^36^ As such, information is required to capture patient transitions into and out of care and how this process can be optimized.

Additionally, there is currently only one group therapy available online (i.e., online drop-in mindfulness group), which may limit accessibility for patients who may not otherwise have the opportunity to attend the clinic due to physical or geographical barriers. To help address this concern, the next steps for the interprofessional team are to provide a larger variety of online groups. Alternating group programs will be available either in person or online.

There is also a need to build an evidence-based guide for shared decision making about treatment priority. For example, some people with depressive symptoms may respond well to the 8-week LITE chronic pain Management program, whereas others may need to complete the cognitive–behavioral therapy program prior to completing the LITE program.

Although discharge criteria from the interprofessional team are clear, the process of discharging patients from the care team can be complex when multiple programs and treatment providers are involved. The team is presently working on discharge considerations to improve this process.

Another opportunity for program optimization lies in leveraging more health providers to refer to lower intensity programs. The interprofessional team currently utilizes the stepped care system as a referral-based option to connect patients to their desired and needed levels of care. It may be advantageous to empower more health professionals, such as nurses and physicians, to use lower intensity programming at their points of contact with patients. For example, nurses could provide patients with pain and mild anxiety who are unable to travel to the clinic on a regular basis with a referral to BounceBack^42^ instead of directing the patient to the interprofessional team for assessment. Using this method, patients will receive quicker access to care that is likely to meet their specific needs.

Infrastructure is also required to support implementation as well as evaluation of stepped care outcomes. This could include the development of technology platforms that facilitate (1) evidence-based clinical decision making; (2) availability and access to interventions; and (3) collection of large data sets for evaluation that will ultimately inform decisions about health care reform at the provincial and federal levels. Such programs are being undertaken in the area of mental health services.^49^

## Conclusion

The adoption of stepped care for the management of chronic pain is promising, but limited data have been collected and initial results have been equivocal due to the implementation of heterogeneous models of stepped care. More studies are needed to evaluate the efficacy and validity of stepped care to manage chronic pain in pain clinic settings. Stepped Care 2.0^17^ has been established and demonstrates promising results to improve individuals’ mental health and satisfaction with receiving care. The pain clinic in the Ottawa General Hospital has implemented a detailed version of Stepped Care 2.0 in efforts to improve chronic pain management. Stepped care has elimited wait list times to access to the interprofessional teams and created a list of available resources at varying levels of intensities that patients could avail of to improve their care. More studies are needed in pain clinic settings to determine whether well-defined steps ultimately translate into greater satisfaction and enhanced outcomes in people who experience chronic pain. Future directions include elucidating various interventions at each step in the model, expanding on and integrating available resources, and introducing electronic support systems that facilitate continuous outcome monitoring as well as stepping of care to improve transitions in care.

## Data Availability

Data are available on request from the corresponding author.
